# Ferulic Acid Enhances Peripheral Nerve Regeneration across Long Gaps

**DOI:** 10.1155/2013/876327

**Published:** 2013-04-16

**Authors:** Sheng-Chi Lee, Chin-Chuan Tsai, Chun-Hsu Yao, Yueh-Sheng Chen, Ming-Chang Wu

**Affiliations:** ^1^Department of Food Science, National Pingtung University of Science and Technology, Pingtung 91201, Taiwan; ^2^Department of Orthopaedics, Pingtung Branch, Kaoshiung Veterans General Hospital, Kaohsiung 91201, Taiwan; ^3^School of Chinese Medicine for Post-Baccalaureate, I-Shou University, Kaohsiung 82445, Taiwan; ^4^Chinese Medicine Department, E-DA Hospital, Kaohsiung 82445, Taiwan; ^5^Lab of Biomaterials, School of Chinese Medicine, China Medical University, Taichung 40402, Taiwan; ^6^Department of Biomedical Imaging and Radiological Science, China Medical University, Taichung 40402, Taiwan

## Abstract

This study investigated the effect of ferulic acid (FA) on peripheral nerve injury. In the *in vitro* test, the effect of FA on viability of Schwann cells was studied. In the *in vivo* test, right sciatic nerves of the rats were transected, and a 15 mm nerve defect was created. A nerve conduit made of silicone rubber tube filled with FA (5 and 25 *μ*g/mL), or saline (control), was implanted into the nerve defect. Results show that the number of proliferating Schwann cells increased significantly in the FA-treated group at 25 *μ*g/mL compared to that in the control group. After 8 weeks, the FA-treated group at 25 *μ*g/mL had a higher rate of successful regeneration across the wide gap, a significantly calcitonin gene-related peptide (CGRP) staining of the lamina I-II regions in the dorsal horn ipsilateral to the injury, a significantly diminished number of macrophages recruited, and a significantly shortening of the latency and an acceleration of the nerve conductive velocity (NCV) of the evoked muscle action potentials (MAPs) compared with the controls. In summary, the FA may be useful in the development of future strategies for the treatment of peripheral nerve injury.

## 1. Introduction

Nerves are not homogenous tissues of monotypic cells. Peripheral nerve regeneration represents a series of highly specialized processes of healing when considered on a cellular level. Numerous investigators have bridged the stumps of a transected peripheral nerve by inserting them in conduits fabricated from a large variety of materials, either biological or artificial [1–4]. These nerve-bridging conduits provide a means for studying the nerve regenerative processes under controlled experimental conditions. However, the investigators' widespread use of a variety of gap lengths may not be able to reveal the real functions of the conduits. In the works of Zhang and Yannas [5], they analyzed a large normalized database from independent investigations, showing that the critical axon elongation, that is, the gap length between the transected sciatic nerve stumps at which the frequency of reconnection is just 50% in an empty silicone rubber nerve conduit, is 9.7 ± 1.8 mm for the rat. Their results implied that the nerve bridge technique can be successfully used to repair short nerve gaps in a rat model. However, the rat peripheral nervous system possesses a high potential for axonal regeneration across short nerve gaps. Therefore, the use of a longer gap more than 10 mm may be necessary in rats to show significant improvements in bridging injured nerves by using different modifications of nerve conduits. Various stimulatory substances such as laminin [6], fibronectin [7], and collagen [8] have been used accompanied with the nerve bridging conduits to modify their internal microenvironment in order to promote the growth of nerves across longer gaps. Recently, administration of herbal medicine has attracted the attention of investigators as a new approach to treat peripheral nerve injuries, such as extraction of Paeoniae alba Radix [9], Chungpa-Juhn [10], Lumbricus [11], and Sanyak [12]. Components in herbs can also accelerate axonal regeneration and target muscle reinnervation, such as quercetin [13], puerarin [14], astragaloside [15], and bilobalide [16].

**Figure 1:**
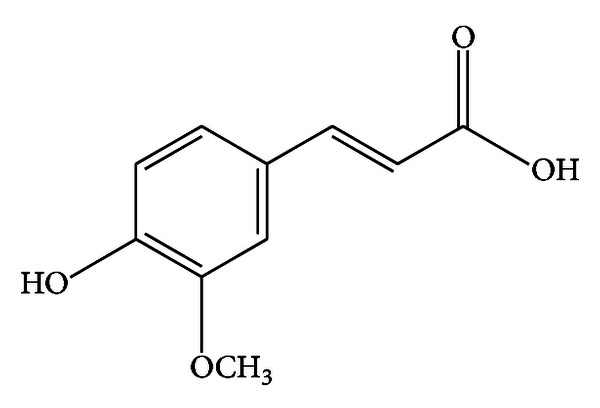
Structure of ferulic acid (FA).

Ferulic acid (FA) is a component of *Angelica sinensis* (Oliv.) Diels, and *Ligusticum chuanxiong* Hort. (Figure 1), was shown to be a free radical scavenger and to have anti-inflammatory and antioxidation effects in a transient middle cerebral artery occlusion model [17]. FA could also decrease the level of intercellular adhesion molecule-1 mRNA and the number of microglia/macrophages and subsequently downregulated inflammation-induced oxidative stress and oxidative stress-related apoptosis after cerebral ischemia/reperfusion injury in rats, suggesting that FA could provide neuroprotection against oxidative stress-related apoptosis [18]. It has been also reported that the FA was better for retinal nerve cell proliferation than that of brain-derived neurotrophic factor [19]. Although the literature shows that FA has versatile biological functions, its nerve growth-promoting effect has never been characterized. Therefore, this study investigated the effect of FA on peripheral nerve injury, using a rat *in vivo* sciatic nerve transection and repair model with a long 15 mm gap and rat Schwann cell line *in vitro*.

## 2. Materials and Methods

### 2.1. Cell Culture and Treatment

 Schwann cells (RSC96) were maintained at 37°C in a humidified atmosphere of 5% CO_2_ and 95% air in DMEM medium supplemented with 5% fetal calf serum, 2 mM HEPES, and 2 mM L-glutamine. The Schwann cells (7 × 10^3^ cells/cm^2^) were then induced to undergo neuronal differentiation by treatment with 5 and 25 *μ*g/mL of FA solution (Product 128708, Sigma-Aldrich, St. Louis, MO, USA) for two days. Cells that had been exposed to the vehicle alone (culture medium only) were the control. Six replicates were used in each study.

### 2.2. Analysis of Cell Viability

 After 48 h of cell incubation, the medium was removed, replaced with 110 *μ*L/well of 5 mg/mL of MTT solution in 1 × PBS, and further incubated in an incubator at 37°C for 4 h. Then, the MTT solution was removed and replaced with 50 *μ*L of DMSO to dissolve the formazan. The color intensity was measured using a microplate reader (ELx800TM, Bio-Tek Instrument, Inc., Winooski, VT, USA) at the absorbance of 550 nm. Data were then expressed as a percent of control level of the optical density within an individual experiment. 

### 2.3. Cell Imaging

 After treating with the FA solution for 48 h, the Schwann cells were washed with PBS twice, fixed in 2% paraformaldehyde for 30 min, and then permeabilized with 0.1% Triton X-100/PBS for 30 min at room temperature. After washing with PBS, TUNEL assay was performed according to the manufacturer's instructions (Boehringer Mannheim). Cells were incubated in TUNEL reaction buffer in a 37°C humidified chamber for 1 h in the dark, then rinsed twice with PBS, and incubated with DAPI (1 mg/mL) at 37°C for 10 min; stained cells were visualized using a fluorescence microscope (Olympus DP70/U-RFLT50, Olympus Optical Co. Ltd., Japan). DAPI-positive and TUNEL-positive cells were counted as live and apoptotic cells, respectively.

### 2.4. Surgical Preparation of Animals

Adult Sprague-Dawley rats underwent placement of silicone chambers. The animals were anesthetized with an inhalational anesthetic technique (AErrane, Baxter, USA). Following the skin incision, fascia and muscle groups were separated using blunt dissection, and the right sciatic nerve was severed into proximal and distal segments. The proximal stump was then secured with a single 9-0 nylon suture through the epineurium and the outer wall of the silicone rubber chamber (1.47 mm ID, 1.96 mm OD; Helix Medical, Inc., Carpinteria, CA, USA). Animals were divided into 3 groups. In group A (*n* = 9), the chambers were filled with normal saline as the controls. In group B (*n* = 9), 5 *μ*g/mL of FA solution was filled in the chambers. Similarly, chambers in group C (*n* = 9) were filled with 25 *μ*g/mL of FA solution, respectively.

The volume of the chamber lumen was approximately 25.5 *μ*L. These fillings, which were in the liquid state, were injected through a micropipette into the lumens by passing the tip of the needle inside the silicone rubber chambers as slowly as possible to prevent the leakage. As we secured the proximal stump into the end of the silicone rubber chamber, the swollen nerve end blocked the opening of the chamber. The chamber was then tilted and the distal stump secured into the other end of the chamber. Both the proximal and distal stumps were secured to a depth of 1 mm into the chamber, leaving a 15 mm gap between the stumps. The muscle layer was reapproximated with 4-0 chromic gut sutures, and the skin was closed with 2-0 silk sutures. All animals were then returned to plastic cages where a sufficient amount of sawdust was present to decrease direct contact of the paralyzed limb of the rat with the floor. These animals were housed in temperature (22°C) and humidity (45%) controlled rooms with 12-hour light cycles, and they had access to food and water *ad libitum*. All animals were maintained in facilities approved by the China Medical University for Accreditation of Laboratory Animal Care and in accordance with current ROC National Science Council of health regulations and standards.

### 2.5. Electrophysiological Techniques

After 8 weeks of regeneration, the animals were reanaesthetized and the silicone rubber conduit was removed to expose their sciatic nerve. The stimulating cathode was a stainless steel monopolar needle, which was placed directly on the sciatic nerve trunk, 5 mm proximal to the transection site. The anode was another stainless steel monopolar needle placed 3 mm proximally to the cathode. Amplitude, area, and nerve conductive velocity (NCV) of the evoked muscle action potentials (MAPs) were recorded from gastrocnemius muscles with microneedle electrodes linked to a computer system (Biopac Systems, Inc., USA). The amplitude and the area under the MAP curve were calculated from the baseline to the maximal negative peak. The NCV was carried out by placing the recording electrodes in the gastrocnemius muscles and stimulating the sciatic nerve proximally and distally to the silicone rubber conduit. The NCV was then calculated by dividing the distance between the stimulating sites by the difference in latency time. All data are expressed as mean ± standard deviation. Statistical comparisons between groups were made by the one-way analysis of variance.

### 2.6. Histological Techniques

After perfusion, as was done for the retrograde labeling, the L4 spinal cord and the distal stump outside the nerve gap were quickly removed and postfixed in the same fixative for 3-4 h. Tissue samples were placed overnight in 30% sucrose for cryoprotection at 4°C, followed by embedding in optimal cutting temperature solution. Samples were kept at −20°C until preparation of 18 *μ*m sections was performed using a cryostat, with samples placed upon poly-L-lysine-coated slide. Immunohistochemistry of frozen sections was carried out using a two-step protocol according to the manufacturer's instructions (Novolink Polymer Detection System, Novocastra). Briefly, frozen sections were required, endogenous peroxidase activity was blocked with incubation of the slides in 0.3%  H_2_O_2_, and nonspecific binding sites were blocked with Protein Block (RE7102; Novocastra). After serial incubation with rabbit- anti-CGRP polyclonal antibody 1 : 1000 (Calbiochem, Germany), Post Primary Block (RE7111; Novocastra), and secondary antibody (Novolink Polymer RE7112), the L4 spinal cord sections were developed in diaminobenzidine solution under a microscope and counterstained with hematoxylin. Similar protocols were applied in the sections from the distal stump except that they were incubated with antirat CD68 1 : 100 (AbD Serotec, Kidlington, UK). Sciatic nerve sections were taken from the middle regions of the regenerated nerve in the chamber. After the fixation, the nerve tissue was postfixed in 0.5% osmium tetroxide, dehydrated, and embedded in Spurr's resin. The tissue was then cut to 5 *μ*m thickness by using a microtome (Leica EM UC6, Leica Biosystems, Mount Waverley, Australia) with a dry glass knife, stained with toluidine blue. 

### 2.7. Image Analysis

All tissue samples were observed under optical microscopy. CGRP immunoreactivity (IR) in dorsal horn in the lumbar spinal cord was detected by immunohistochemistry. The immunoproducts were confirmed positive labeled if their density level was over five times background levels. Under a 100x magnification, the ratio of area occupied by positive CGRP-IR in dorsal horn ipsilateral to the injury following neurorrhaphy relative to the lumbar spinal cord was measured using an image analyzer system (Image-Pro Lite, Media Cybernetics, USA) coupled to the microscope. 

As counting the myelinated axons, at least 30 to 50 percent of the sciatic nerve section area randomly selected from each nerve specimen at a magnification of 400x was observed. The axon counts were extrapolated by using the area algorithm to estimate the total number of axons for each nerve. Axon density was then obtained by dividing the axon counts by the total nerve areas. Similarly, the density of macrophage was determined by dividing the macrophage counts by the total nerve areas. All data are expressed as mean ± standard deviation. Statistical comparisons between groups were made by one-way analysis of variance with Scheffe's test.

## 3. Results

### 3.1. Cell Viability

**Figure 2:**
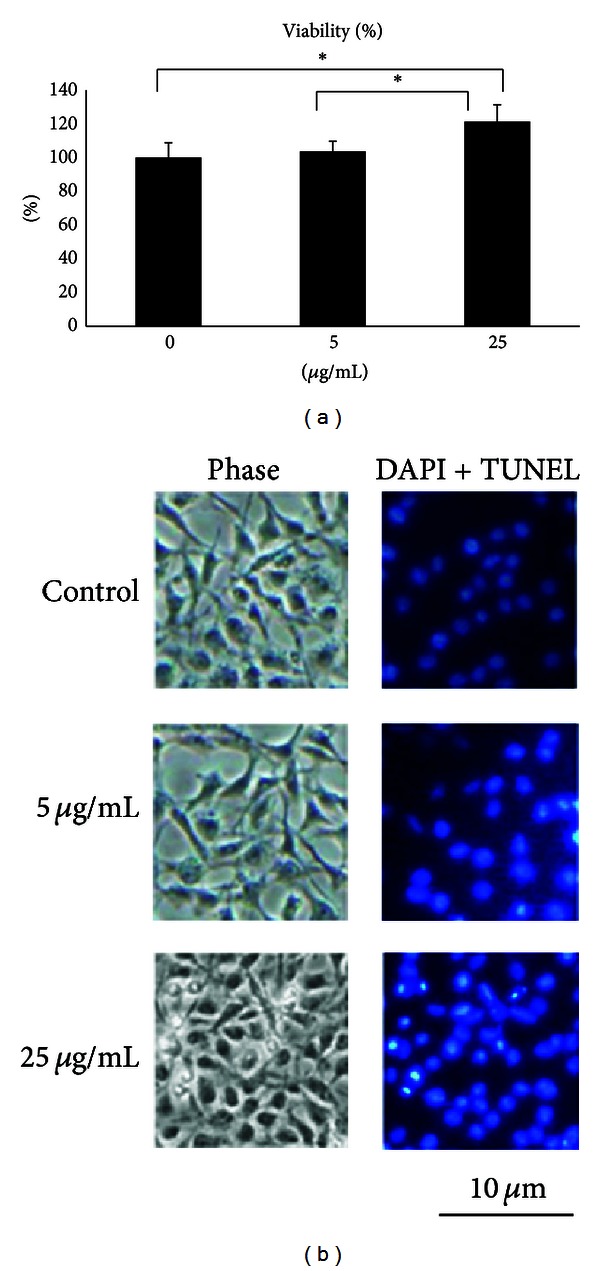
(a) Quantification of viability of Schwann cells treated with ferulic acid relative to controls. Values are means ± S.E.M. *P < 0.05 indicates a significant difference from other groups. (b) Nuclei of Schwann cells characterized by DAPI and TUNEL assays and investigated via fluorescent microscopy.

When RSC96 cells were treated with FA at 25 *μ*g/mL for 24 h, cell viability significantly increased to 121% of control (*P* < 0.05, Figure 2(a)). Cell imaging also strongly supports the beneficial effect of FA in proliferation of Schwann cells that increases DAPI positive cells, and no TUNEL positive cells were seen in both the FA-treated groups compared to the controls (Figure 2(b)). Taken together, these data indicate that treatment with FA promotes viability of Schwann cells.

### 3.2. Electrophysiological Measurements

**Figure 3:**
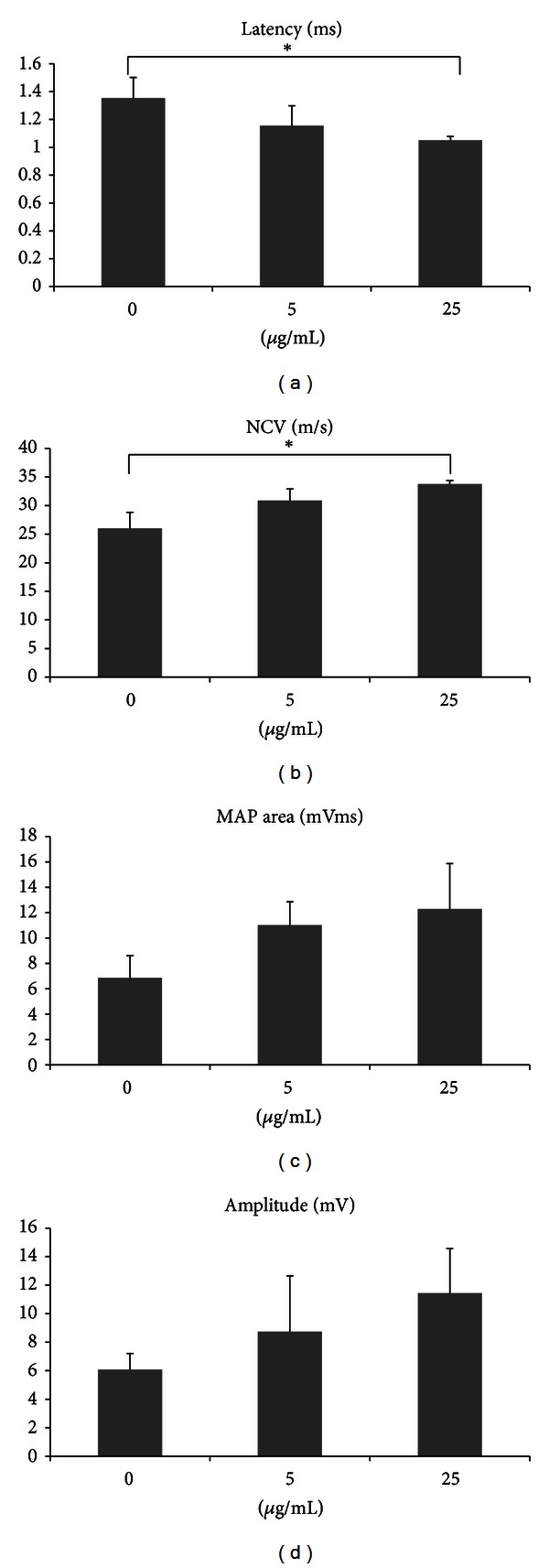
Analysis of evoked MAPs, including (a) latency, (b) NCV, (c) peak amplitude, and (d) area under the MAP curves. *P < 0.05 indicates significant difference from other groups.

Rats having regenerated cables within the bridging conduits presented a triphasic wave of MAP indicating successful reinnervation of the gastrocnemius muscle by the regenerating sciatic nerve. The comparison of latency or NCV of the MAP waves showed significant differences between FA-treated rats at 25 *μ*g/mL and controls (*P* < 0.05, Figures 3(a) and 3(b)). The FA-treated animals had a dramatic shortening of the latency and an acceleration of the NCV compared with the controls. The FA-treated animals also had a relatively larger area and amplitude of the MAP wave compared with the controls, though their differences did not reach the significant level at *P* = 0.05 (Figures 3(c) and 3(d)).

### 3.3. CGRP-IR in the Dorsal Horn following Injury

**Figure 4:**
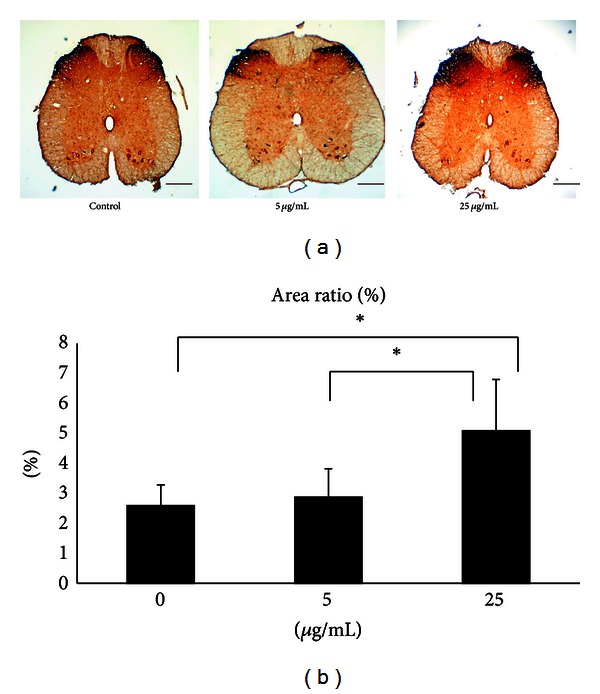
(a) Photomicrographs demonstrating CGRP-IR in dorsal horn in the lumbar spinal cord after injury of controls and FA-treated animals. Scale bars = 200 *μ*m. (b) Note the significantly increased CGRP-IR area ratios in FA-treated animals at 25 *μ*g/mL compared to those at 5 *μ*g/mL and the controls. *P < 0.05 indicates significant difference from other groups.

Immunohistochemical staining showed that CGRP-labeled fibers were seen in the area of lamina III–V and lamina I-II regions in the dorsal horn ipsilateral to the injury in all of the rats (Figure 4(a)) in which the FA-treated rats at 25 *μ*g/mL had a significantly higher CGRP expression compared with the FA-treated rats at 5 *μ*g/mL and the controls (*P* < 0.05, Figure 4(b)). 

### 3.4. Macrophages Recruited in the Distal Nerve Ends

**Figure 5:**
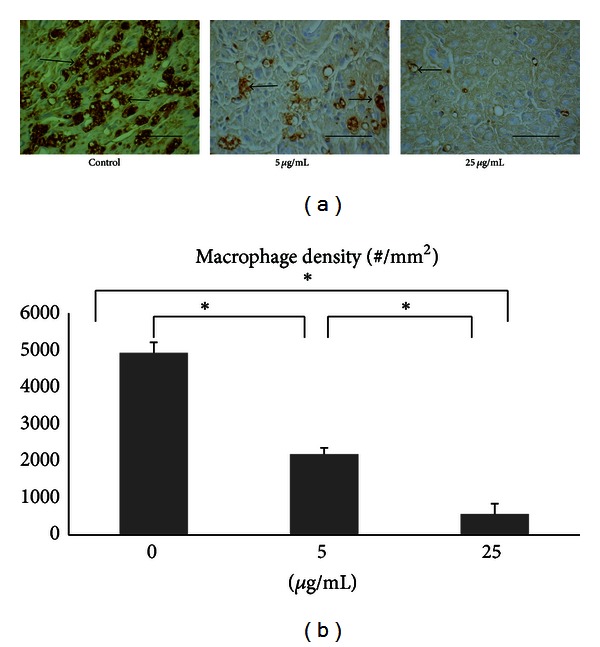
(a) Photomicrographs demonstrating antirat CD68 immunoreactivity in macrophages (arrows) from cross sections of distal nerve cables of controls and FA-treated animals. Scale bars = 100 *μ*m. (b) Note the dramatically decreased density of macrophages with the concentration of FA. *P < 0.05 indicates significant difference from other groups.

 Within distal portions of the transection gap zone we identified clusters of macrophages labeled with CD68. Obviously, the FA could alleviate local inflammatory conditions in the regenerated nerves in which the density of macrophage was decreased as the concentration of FA was increased (Figure 5(a)). Specially, a significantly lower density of macrophages was recruited into the nerve stumps in the FA-treated animals at 25 *μ*g/mL compared to that at 5 *μ*g/mL and the controls (*P* < 0.05, Figure 5(b)). This result indicated that deliberate superimposition of FA in the bridging conduits could dramatically suppress influx of macrophages in injured nerves.

### 3.5. Sciatic Nerve Regeneration

**Figure 6:**
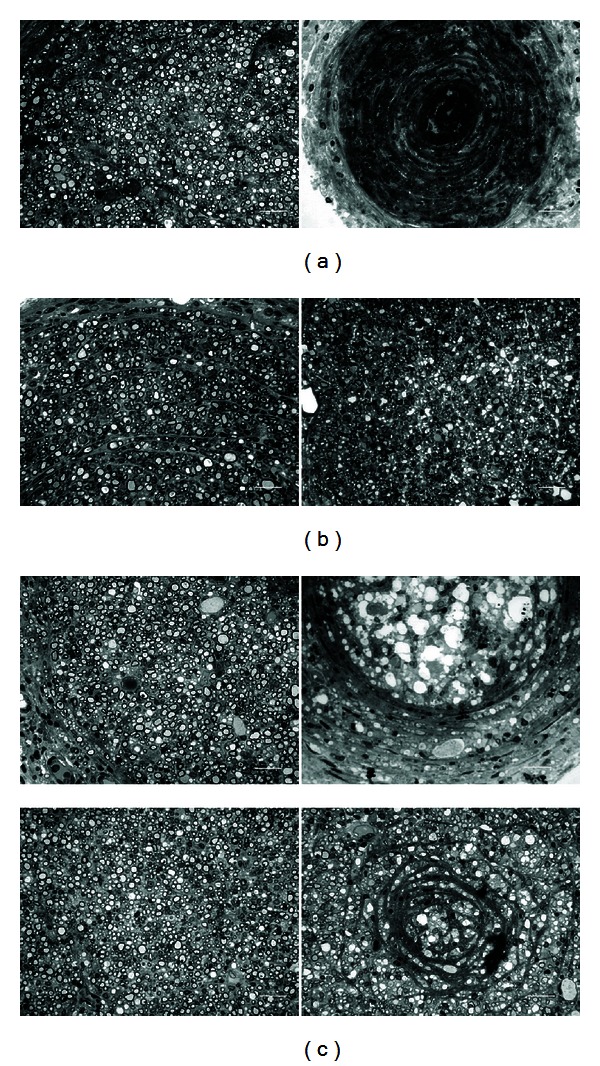
Photomicrographs showing regenerated nerve cross sections of (a) controls and FA-treated animals at (b) 5 *μ*g/mL and (c) 25 *μ*g/mL. Scale bars = 20 *μ*m.

 Features of axon regeneration in bridging conduits have largely been described at the light and electron microscopic level. Usually, connective tissue regenerative bridges populated by a core of myelinated axons surrounded by layers of perineurial and epineurial cells could be identified. In this study, we found that two of nine conduits in the control group developed regenerative bridges by the 8-week end point (Figure 6(a)). Only one of them had evidence of axon regrowth (number: 3592) and penetration into the bridge. In conduits exposed to FA at 5 *μ*g/mL, two of nine had bridges (Figure 6(b)), and the mean axonal number was 1627 (range: 1600–1653). By comparison, four of nine rats treated with 25 *μ*g/mL of FA developed the regenerative bridges (Figure 6(c)) and three of which contained a sizeable population of myelinated axons (mean axon number was 2437; range: 691–3963). 

## 4. Discussion

Most of the studies in the literature associated with the nerve regeneration using herbal medicine to repair injured rat nerves have reported that the interstump gap lengths used were shorter than 10 mm. A 10 mm gap is considered the critical gap length within a tubular prosthesis above which neural components usually will not cross the gap when no growth substances are distributed in the chambers at the time of implantation [20]. Therefore, to demonstrate the real effect of herbal medicine on nerve regeneration we chose a 15 mm gap in the rat transaction model. 

 Before examining the *in vivo* action of FA on nerve regeneration, we first found that FA could increase Schwann cell viability in a dose-dependent fashion *in vitro*. Schwann cells are the principal glia as well as myelinating cells of the peripheral nervous system, which are involved in many important aspects of peripheral nerve regeneration, such as secreting neurotrophic factors and physically supporting regenerating axons [21]. Therefore, the FA should have significant nerve growth-promoting potential. The *in vivo* study supports and extends these initial *in vitro* findings by showing that FA could enhance formation of regenerated nerve cables in the bridging conduits, especially in the group of FA at 25 *μ*g/mL (4 of 9). By comparison, the complete interruption of regenerative bridge formation was dramatic in the controls (7 of 9) and the FA-treated rats at 5 *μ*g/mL (7 of 9). In addition, histological observations of regenerated nerve also strongly support the beneficial effect of FA in axonal regrowth that only one of the regenerated cables in the controls showing myelinated axons. Similar to the morphometric analyses, the FA effect was also observed in the MAP measurements. For example, the FA group at 25 *μ*g/mL showed bundles of myelinated axons in morphometric analysis with a relatively larger amplitude, area, NCV, and shorter latency during electrophysiological assay as compared to those in the other two groups. To address how regenerative events might fare in bridges exposed to FA, we also examined CGRP-labeled components in the spinal cord sections. Unlike control or low-dose FA infused sections, the FA-treated rats at 25 *μ*g/mL had a dramatic high CGRP expression. The CGRP is crucial for interactions between axons and Schwann cells during peripheral nerve regrowth [22] and plays important roles in nerve function and repair when axons are severed [23]. Taken together, these data indicate that treatment with FA at 25 *μ*g/mL could promote functional recovery by accelerating axonal regeneration after sciatic nerve transaction in the rat.

 Finally, in this study, a surface marker, CD68, was employed to characterize macrophages participating in nerve regeneration. It was found that population of macrophages was significantly diminished as the dosage of FA was increased. Macrophages have been reported, playing an important role during early nerve regeneration [24]. Within days of peripheral nerve injury, activated macrophages begin tasks of debris removal, secretion of growth factors, and remodeling of the extracellular matrix of the distal nerve stump. However, at later stage of nerve regeneration inflammation caused by macrophages becomes harmful since nitric oxide (NO) generated by inducible nitric oxide synthase (iNOS) can collapse growth cones and cause axonal degeneration [25]. Since this study focused on late regenerative events associated with axon growth across a critical defect gap, population of macrophages suppressed by administering FA in the bridging conduits was beneficial to the regenerating axons. 

## 5. Conclusions

Our data demonstrates that FA appears to promote peripheral nerve regeneration across a 15 mm critical defect gap in the rat sciatic nerve injury model. Suppression of macrophages by FA at the site of peripheral nerve injury may contribute to its nerve growth-promoting capability.
